# Loss of Sirt3 Limits Bone Marrow Cell-Mediated Angiogenesis and Cardiac Repair in Post-Myocardial Infarction

**DOI:** 10.1371/journal.pone.0107011

**Published:** 2014-09-05

**Authors:** Heng Zeng, Lanfang Li, Jian-Xiong Chen

**Affiliations:** Department of Pharmacology and Toxicology, University of Mississippi Medical Center, Jackson, Mississippi, United States of America; University of Cincinnati, College of Medicine, United States of America

## Abstract

Sirtuin-3 (Sirt3) has a critical role in the regulation of human aging and reactive oxygen species (ROS) formation. A recent study has identified Sirt3 as an essential regulator of stem cell aging. This study investigated whether Sirt3 is necessary for bone marrow cell (BMC)-mediated cardiac repair in post-myocardial infarction (MI). *In vitro*, BMC-derived endothelial progenitor cells (EPCs) from wild type (WT) and Sirt3KO mice were cultured. EPC angiogenesis, ROS formation and apoptosis were assessed. *In vivo*, WT and Sirt3 KO mice were subjected to MI and BMCs from WT and Sirt3 KO mice were injected into ischemic area immediately. The expression of VEGF and VEGFR2 was reduced in Sirt3KO-EPCs. Angiogenic capacities and colony formation were significantly impaired in Sirt3KO-EPCs compared to WT-EPCs. Loss of Sirt3 further enhanced ROS formation and apoptosis in EPCs. Overexpression of Sirt3 or treatment with NADPH oxidase inhibitor apocynin (Apo, 200 and 400 microM) rescued these abnormalities. In post-MI mice, BMC treatment increased number of Sca1^+^/c-kit^+^ cells; enhanced VEGF expression and angiogenesis whereas Sirt3KO-BMC treatment had little effects. BMC treatment also attenuated NADPH oxidase subunits p47^phox^ and gp91^phox^ expression, and significantly reduced ROS formation, apoptosis, fibrosis and hypertrophy in post-MI mice. Sirt3KO-BMC treatment did not display these beneficial effects. In contrast, Sirt3KO mice treated with BMCs from WT mice attenuated myocardial apoptosis, fibrosis and improved cardiac function. Our data demonstrate that Sirt3 is essential for BMC therapy; and loss of Sirt3 limits BMC-mediated angiogenesis and cardiac repair in post-MI.

## Introduction

Sirtuins belong to a highly conserved family (Sirtuin 1–7) of histone/protein deacetylases and its activity is closely associated with the prolong lifespan of organisms such as yeast, worms and flies as well as mammalian [1]. Sirtuins mediate histone protein post-translational modification by coupling lysine deacetylation to NAD^+^ hydrolysis [2], [3]. Sirtuins have critical roles in the regulation of various cell functions, including cardiomyocytes and endothelial cells [4]–[7]. Sirtuins have been shown to involve in biological functions related to cell growth, aging, stress tolerance as well as cell metabolism [1], [8]. Accumulating evidence indicates an important role of Sirt3 in the genetic control of human aging. Older individuals have about 40% reduced Sirt3 levels when compared with younger subjects, and the health benefits of older patients are accompanied by the elevation of Sirt3 levels [9]. Further, increased levels of Sirt3 are associated with an extended lifespan of man [10], [11]. Calorie restriction has been shown to increase Sirt3 expression and improve cardiovascular function [12]. Moreover, Sirt3 KO mice are resistant to the protective effects of calorie restriction against oxidative damage [13]. In contrast, overexpression of Sirt3 blocks cardiac hypertrophy by suppressing reactive oxygen species (ROS) formation. Knockout of Sirt3 in mice promotes angiotensin II-induced cardiac hypertrophy [14], [15].

Myocardial infarction (MI) has been shown to induce rapid mobilization of bone marrow derived cells (BMCs) such as endothelial progenitor cells (EPCs), mesenchymal stromal cells and pluripotent very small embryonic-like cells into circulation and home to sites of ischemic area, and promote neovascularization [16], [17]. BMC treatment has been shown to lead to a reduction of infarct size and improvement of cardiac function in post-MI [18]. BMCs have been identified as promising candidates for use as cellular therapies for cardiac regeneration and repair in post-MI. Sirt3 is a key regulator of mitochondria ROS formation and telomere length [14], [19]. Recent studies highlight the important role of Sirt3 in BMC-derived stem cells [20], [21]. Sirt3 expression is significantly reduced in aged hematopoietic stem cells (HSCs). Moreover, upregulation of Sirt3 expression improves their regenerative capacity in aged HSCs [21]. Consistent with these findings, our recent study also shows that loss of Sirt3 attenuates apelin-overexpressing BMC-mediated improvement of cardiac repair and function in post-MI [20]. However, the direct roles of Sirt3 in the stem cell therapy-mediated cardiac repair and functional recovery in post-MI remain undefined. In this study, we tested our hypothesis that Sirt3 in the BMCs is essential for the stem cell therapy-mediated angiogenesis and cardiac repair in post-MI.

Using BMCs and EPCs from wild type (WT) mice and Sirt3 knockout (Sirt3KO) mice, this study was to determine: (1) whether loss of Sirt3 in EPCs reduces angiogenic growth factor expression and blunts their proangiogenic and anti-apoptotic capacities; (2) whether loss of Sirt3 in BMCs dampens BMC-mediated angiogenesis and cardiac repair in post-MI mice.

## Materials and Methods

### Ethics Statement

All procedures conformed to the Institute for Laboratory Animal Research Guide for the Care and Use of Laboratory Animals and were approved by the University of Mississippi Medical Center Animal Care and Use Committee (Protocol ID: 1280). The investigation conforms to the Guide for the Care and Use of Laboratory Animals published by the US National Institutes of Health (NIH Publication No. 85–23, revised 1996).

### Surgical procedures

Global Sirt3 knockout mice and wild type control of Sirt3 mice (WT) was purchased from Jackson laboratory (Bar Harbor, ME) and breeding by our laboratory. Male Sirt3KO and WT mice at age 12 weeks were used for the experiments. Male mice were anesthetized with ketamine (100 mg/kg) plus xylazine (15 mg/kg), intubated, and artificially ventilated with room air. Adequate anesthesia was monitored by toe pinch. Myocardial infarction was achieved by ligation of the left anterior descending coronary artery (LAD). Sham controls underwent surgery without the LAD [22], [23], [24]. After induction of myocardial ischemia (IS), mice were intramyocardial injected with fresh donor bone marrow–derived mononuclear cells (1×10^7^cells) immediately [20]. Two weeks after myocardial infarction, mice were sacrificed by cervical dislocation under anesthesia with isoflurane.

### Analysis of APJ^+^/Sca1^+^/c-kit^+^ cells in the heart

Heart tissue sections (8 µm) from injected area of ischemia were incubated with Sca1 and c-kit (1∶200 Santa Cruz, CA) antibodies overnight. Sca1 was visualized using FITC labeled goat anti-mouse IgG antibodies; c-kit was visualized with Fluorolink Cy3 labeled goat anti-mouse IgG antibodies. Myocardial Sca1^+^/c-kit^+^ cells in the injected area were assessed by counting the number of positive cells per 100 nuclei [22], [25].

### Western analysis of Sirt3, VEGF, VEGFR2, eNOS, Akt, p47^phox^, gp91^phox^, CXCR4, beclin-1 and LC3-I/II expression

The hearts or EPCs were harvested and homogenized in lysis buffer for Western blot analysis. Total protein concentrations were determined using a BCA protein assay kit (Pierce Co, IL). Fifteen µg of protein were subjected to SDS-PAGE on 10% polyacrylamide gels and transferred to a nitrocellulose membrane. The blot was probed with Sirt3, VEGFR2, Akt and eNOS (1∶1000, Cell Signaling, MA), VEGF, CXCR-4, gp91^phox^, p47^phox^, LC3-I/II and beclin-1 (1∶1000, Santa Cruz, CA) antibodies. The membranes were then washed and incubated with a secondary antibody coupled to horseradish peroxidase and densitometric analysis was carried out using image acquisition and analysis software (TINA 2.0).

### Analysis of myocardial capillary and arteriole densities

Eight-micrometer sections were cut and incubated with fluorescerin-labeled Isolectin B4 (1∶200; IB4, Molecular Probe, Invitrogen, OR) and Cy3-conjugated anti-α smooth muscle actin (SMA) (1∶100; Sigma). The number of capillaries (IB4-positive EC) in the border zone area was counted and expressed as capillary density per square millimeter (mm^2^) of tissue. Myocardial arteriole density was measured using image analysis software (Image J, NIH, MD) [22], [24].

### Myocardial apoptosis and ROS formation

Heart tissue sections were stained with transferase deoxyuridine nick end labeling (TUNEL) following the manufacturer's instructions (Promega, WI). Apoptosis was indexed by counting TUNEL positive cells per 100 nuclei in the infarcted area [22], [23]. ROS formation in the infarcted area was measured and quantified by staining with DHE as previously described [26].

### Hemodynamic measurements

Experimental mice were anesthetized with ketamine (100 mg/kg) plus xylazine (15 mg/kg), intubated and artificially ventilated with room air. A 1.4-Fr pressure–conductance catheter (SPR-839, Millar Instrument, TX) was inserted into the left ventricle (LV) to record baseline cardiac hemodynamics of the hearts [22], [25].

### Heart weight to body weight ratio (HW/BW) and fibrosis

Cardiac hypertrophy was assessed by measuring heart-to-body weight ratio at 14 days post-myocardial ischemia. Cardiac β-myosin heavy chain (β-MHC) (1∶1000, abcam, MA) and atrial natriuretic peptide (ANP) (1∶1000, Santa Cruz, CA) expression were examined by western blot analysis. Cardiac fibrosis was stained with Masson's trichrome (MT, Sigma, MO) and quantified by measuring the blue fibrotic area [24].

### Cultured EPC proliferation, tuber formation and apoptosis

Wild type (WT) mice and Sirt3 knockout mice were sacrificed by cervical dislocation under anesthesia with isoflurane. BM–derived EPC were obtained by flushing the tibias and femurs with 10% FBS EGM. EPC was isolated and cultured from femur and tibia bone marrow of WT and Sirt3KO mice as described previously [22], [23]. Two EPC markers, IB4 (1∶50 dilute) and CD34 (1∶200 dilute), were used for EPC identification by immunohistochemistry. Deficiency of Sirt3 in the EPCs was verified by western blot analysis. For the cell proliferation measurement, EPCs were cultured in 10%FBS EGM for 72 hours. The proliferative capacity of cultured EPCs was assayed using a cell proliferation (MTT) kit according to the manufacturer's instructions (Roche Diagnostic Corp., IN, USA) [27], [28]. In the apoptosis study, EPCs apoptosis was induced by exposure of cultured EPCs to serum-free medium for 48 hours. The number of apoptotic cells was then examined by counting TUNEL positive cells per 100 nuclei in cultured EPCs.

### Measurement of intracellular ROS formation in cultured EPCs

Intracellular ROS were determined by oxidative conversion of cell permeable chloromethyl-2′,7′-dichlorodihydrofluorescein diacetate (CM-H_2_DCFDA, Molecular Probes, OR) to fluorescent dichlorofluorescein (DCF). Briefly, BMCs cultured in 2 well chamber slides were incubated with 10 µM CM-H_2_DCFDA in PBS for 30 minutes. DCF fluorescence was measured over the whole field of vision using an EVOS fluorescence microscope connected to an imaging system as previously described [29], [30].

### BM colony-forming cell assay

BM–derived mononuclear cells were isolated from WT and Sirt3 KO mice. BMCs (105 cells per dish) were then seeded in 2% methylcellulose medium. After 7 days of incubation, BMC colony formation and colony number were scored under phase-contrast microscopy [31].

### Statistical analysis

The results were expressed as the mean ± SD. Statistical analysis was performed using one way ANOVA followed by post hoc multiple comparisons test. Significance was set at *P*<0.05.

## Results

### Loss of Sirt3 in BMCs reduces c-kit^+^/Sca1^+^ cells in post-MI mice

**Figure 1:**
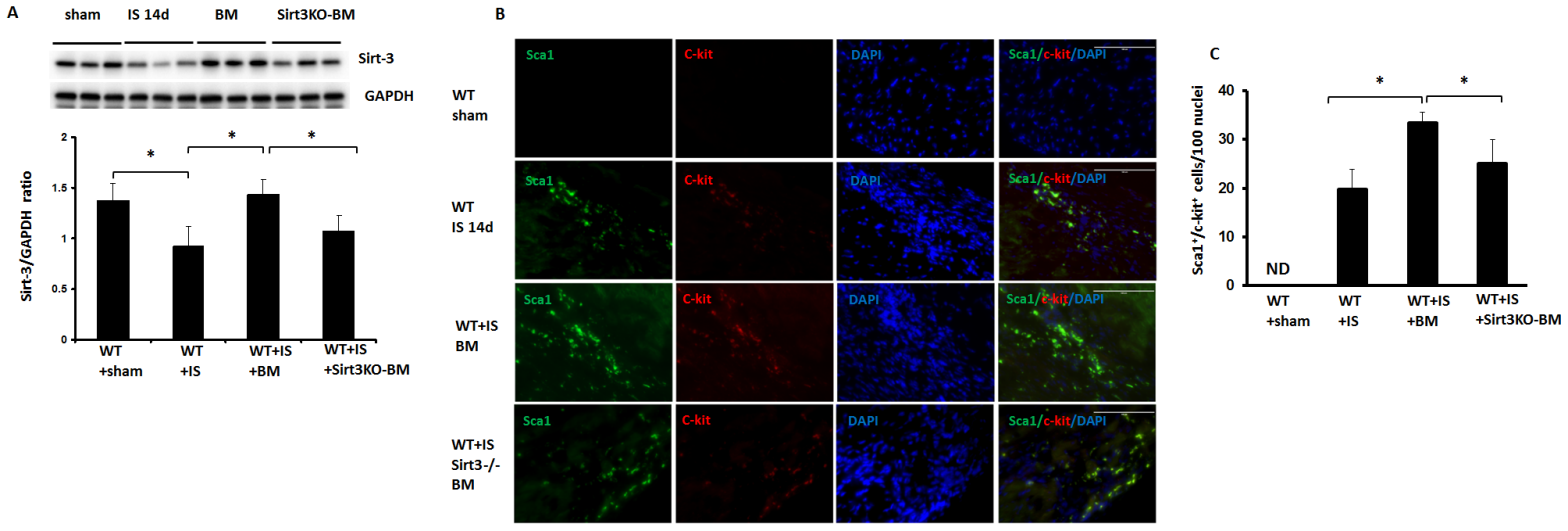
Loss of Sirt3 reduces c-kit^+^/Sca1^+^ cell in post MI mice. **A**. Western blot analysis revealing that Sirt3 expression was significantly reduced in post-MI mice. BMC treatment significantly increased Sirt3 expression compared to post-MI mice. Loss of Sirt3 blunted BMC-mediated upregulation of Sirt3. n = 6 mice, *p<0.05. **B**. Immunofluorescence images showing co-localization of Sca1 and c-kit in the border zone of ischemic mouse hearts. Sca1 was stained with mouse Sca1 antibody (green, 10X). c-kit was stained with rabbit c-kit antibody (red, 10X) and nuclei were stained with DAPI (blue, 10X). **C**. Quantitative analysis of Sca1^+^/c-kit^+^ cells demonstrating that the number of Sca1^+^/c-kit^+^ cells was increased at 14 days of post-MI mice. BMCs significantly increased Sca1^+^/c-kit^+^ cells compared to saline treatment. Treatment with Sirt3KO-BMC had a significant lower number Sca1^+^/c-kit^+^ cells in infracted heart compared to the BMC treated mice. All data represent mean ± SD; n = 5, *p<0.05. ND =  not detected.

We first examined whether Sirt3 expression is altered in the hearts of post-MI mice. As shown in Fig 1A, there was a significant reduction of Sirt3 expression in the hearts of post-MI mice. Interestingly, BMC treatment led to a significant increase in Sirt3 expression in post-MI mice when compared with control post-MI mice (**Fig 1A**). Treatment with Sirt3 KO-BMCs also increased Sirt3 expression in the ischemic hearts of WT mice, but it was significantly less than WT-BMC treatment (Fig 1A).

We next examined whether BMC treatment increases vascular progenitor cells in the infarcted hearts. The number of c-kit^+^/Sca1^+^ cells was evaluated at 14 days after BMC intramyocardial injection. As shown in **Fig 1 B and C**, the number of c-kit^+^/Sca1^+^ cells was increased in the mouse infarcted heart after 14 days of MI, however, no c-kit^+^/Sca1^+^ cell was detected in the non-ischemic sham control mice. BMC treatment significantly increased the number of c-kit^+^/Sca1^+^ cells compared to saline treatment (**Fig 1 B and C**). Injection with Sirt3KO-BMCs had a significant lower number of c-kit^+^/Sca1^+^ cells in infarcted hearts in comparison with BMC treated mice (**Fig 1 B and C**). To determine whether the increased c-kit^+^/Sca1^+^ cells came from the donor or the recipient, mice were intramyocardial injected with GFP^+^-BMCs or GFP^+^-Sirt3KO-BMCs. No GFP^+^-BMCs or GFP^+^-Sirt3KO-BMCs were found in hearts of post-MI mice after 14 and 28 days of BMC treatment (data not shown), indicating these cells may came from recipient but not from donor.

### Loss of Sirt3 increases ROS formation and apoptosis in EPCs

**Figure 2:**
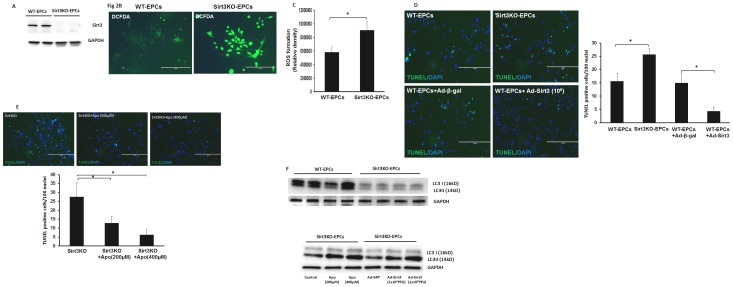
Loss of Sirt3 increases ROS formation and apoptosis in EPCs. **A**. Western blot analysis showing that Sirt3 expression was absent in EPCs isolated from Sirt3KO mice. n = 2 mice. **B and C**. Representative images and quantification of intracellular ROS formation measured by CM-H_2_DCFDA staining in cultured EPCs. ROS formation was significantly increased in cultured EPCs of Sirt3KO mice when compared with that of WT mice (n = 4 mice, *p<0.05). **D and E**. Representative images and quantification of TUNEL positive cell in cultured EPCs. Apoptotic cells were identified by TUNEL staining (green, 20x). Cell apoptosis was significantly increased in cultured EPCs of Sirt3KO mice compared to that of WT mice. Infection of WT-EPC with Ad-Sirt3 (10^6^ PFU) significantly reduced EPC apoptosis (n = 6 mice, *p<0.05). Treatment of Sirt3KO-EPC with NADPH oxidase inhibitor apocynin (Apo, 200 and 400 µM significantly reduced EPC apoptosis (n = 6 mice, *p<0.05). **F**. Western blot analysis showing that the basal levels of autophagy gene LC3 II expression were dramatic reduced in the Sirt3KO-EPCs when compared with WT-EPCs. Treatment of Sirt3KO-EPCs with Apo 200 and 400 µM or infection of Sirt3KO-EPCs with Ad-Sirt3 resulted in an increase in LC3 II expression (n = 3 mice).

Knockout of Sirt3 in BM-derived HSCs has been reported to lead to a 50% reduction in self- renewal compared to WT mice after serial transplantations [21]. We then examined whether loss of Sirt3 affected EPCs function *in vitro*. Our western blot analysis confirmed that Sirt3 expression was absent in EPCs isolated from Sirt3KO mice (**Fig 2A**). In cultured EPCs, there was a significant increase in ROS formation in Sirt3KO-EPCs when compared with WT-EPCs (**Fig 2 B and C**). Moreover, knockout of Sirt3 in EPCs resulted in a significant increase in stress-induced cell apoptosis. Overexpression of Sirt3 significantly reduced stress-induced EPC apoptosis (**Fig 2 D**). Moreover, treatment of Sirt3KO-EPCs with NADPH oxidase inhibitor apocynin (Apo, 200 and 400 microM) attenuated EPC apoptosis in a dose-dependent manner (**Fig 2E**). Interestingly, autophagy marker LC3-I/II was reduced in Sirt3KO-EPCs. Treatment of Sirt3KO-EPCs with Apo (200 and 400 microM) resulted in an increase in LC3-II levels. Furthermore, overexpression of Sirt3 rescued impairment of LC3-II expression in Sirt3KO-EPCs (**Fig 2F**).

### Loss of Sirt3 reduces angiogenic growth factor expression and angiogenesis in EPCs

**Figure 3:**
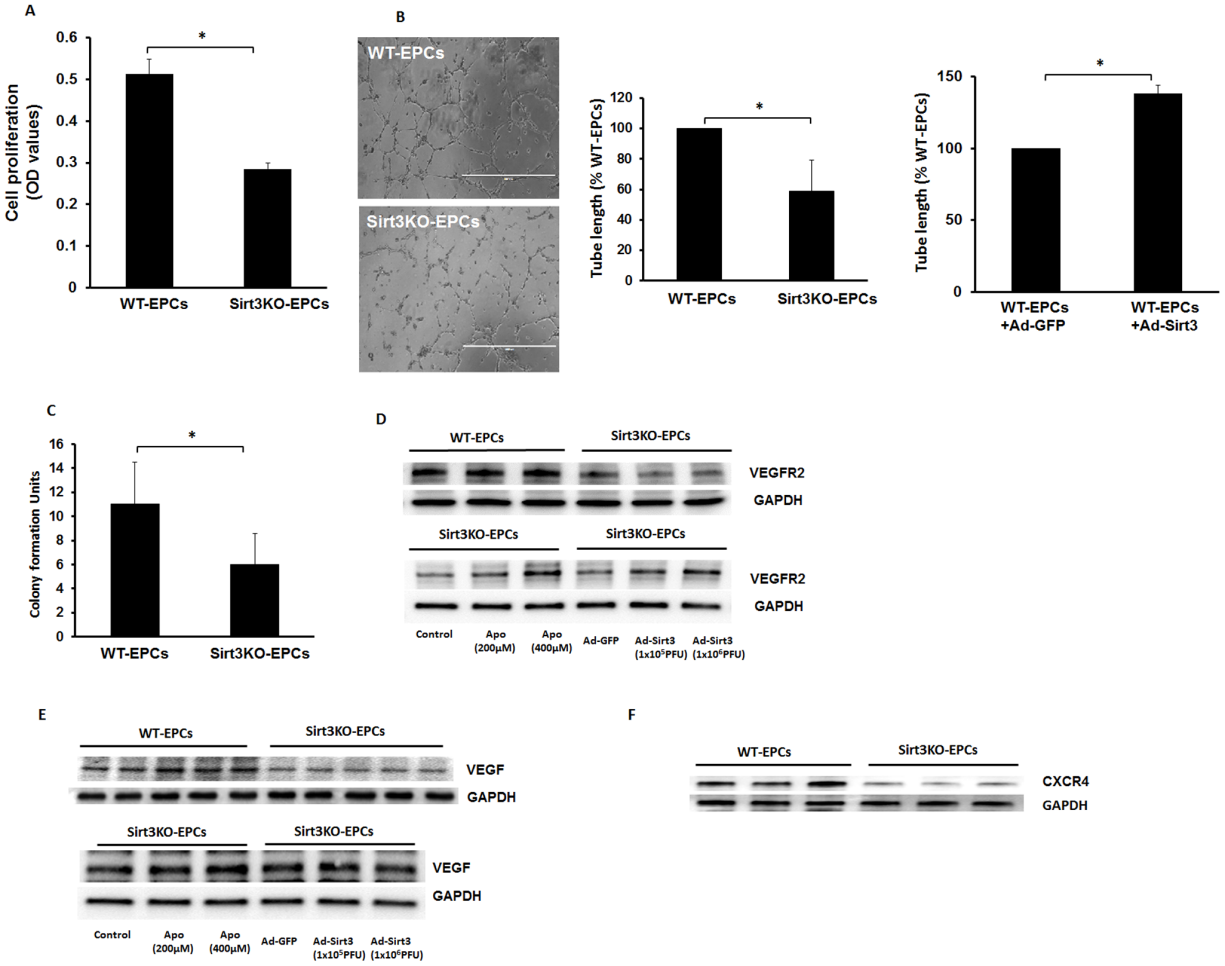
Loss of Sirt3 impairs VEGF/VEGFR2 expression and angiogenesis in EPCs. **A**. Cell proliferation was measured by MTT assay. The proliferative rate of EPCs was significantly reduced in cultured EPCs of Sirt3KO mice compared to that of WT mice (n = 4 mice, *p<0.05). **B**. EPC tube formation was significantly reduced in EPCs lack of Sirt3 when compared with control EPCs. Overexpression of Sirt3 significantly increased EPC tube formation (n = 4–6 mice, *p<0.05). **C**. BMC colony formation units. EPC colony formation was significantly reduced in Sirt3KO-EPCs when compared with WT-EPCs (n = 6 mice, *p<0.05). **D and E**. Western blot analysis showing that the basal levels of VEGF and VEGFR2 were significantly reduced in Sirt3KO-EPCs (n = 3 mice). Treatment of Sirt3KO-EPCs with NADPH oxidase inhibitor apocynin 200 and 400 µM or infection of Sirt3KO-EPCs with Ad-Sirt3 increased levels of VEGF and VEGFR2 expression (n =  3 mice). **F**. Western blot analysis showing that the basal levels of CXCR-4 expression were dramatic reduced in the Sirt3KO-EPCs (n = 3 mice).

Similarly, the proliferation of EPCs was significantly reduced in Sirt3KO-EPCs compared with WT-EPCs (**Fig 3A**). Loss of Sirt3 in EPCs resulted in a significant decrease in tube formation when compared with WT-EPCs. In contrast, overexpression of Sirt3 in WT-EPCs significantly enhanced EPC tube formation (**Fig 3B**). EPC colony formation was significantly lower in Sirt3KO-EPCs than WT-EPCs (**Fig 3C**). In addition, VEGF and VEGFR2 levels were reduced in Sirt3KO-EPCs; whereas treatment with Apo (200 and 400 microM) or overexpression of Sirt3 rescued impaired VEGF/VEGFR2 expression in Sirt3KO-EPCs (**Fig 3D and E**). Intriguingly, basal levels of CXCR-4, an EPC recruitment factor, were significantly decreased in Sirt3KO-EPCs when compared with WT-EPCs (**Fig 3F**).

### Loss of Sirt3 in BMCs increases p47^phox^ and gp91^phox^ expression in the heart of post-MI

**Figure 4:**
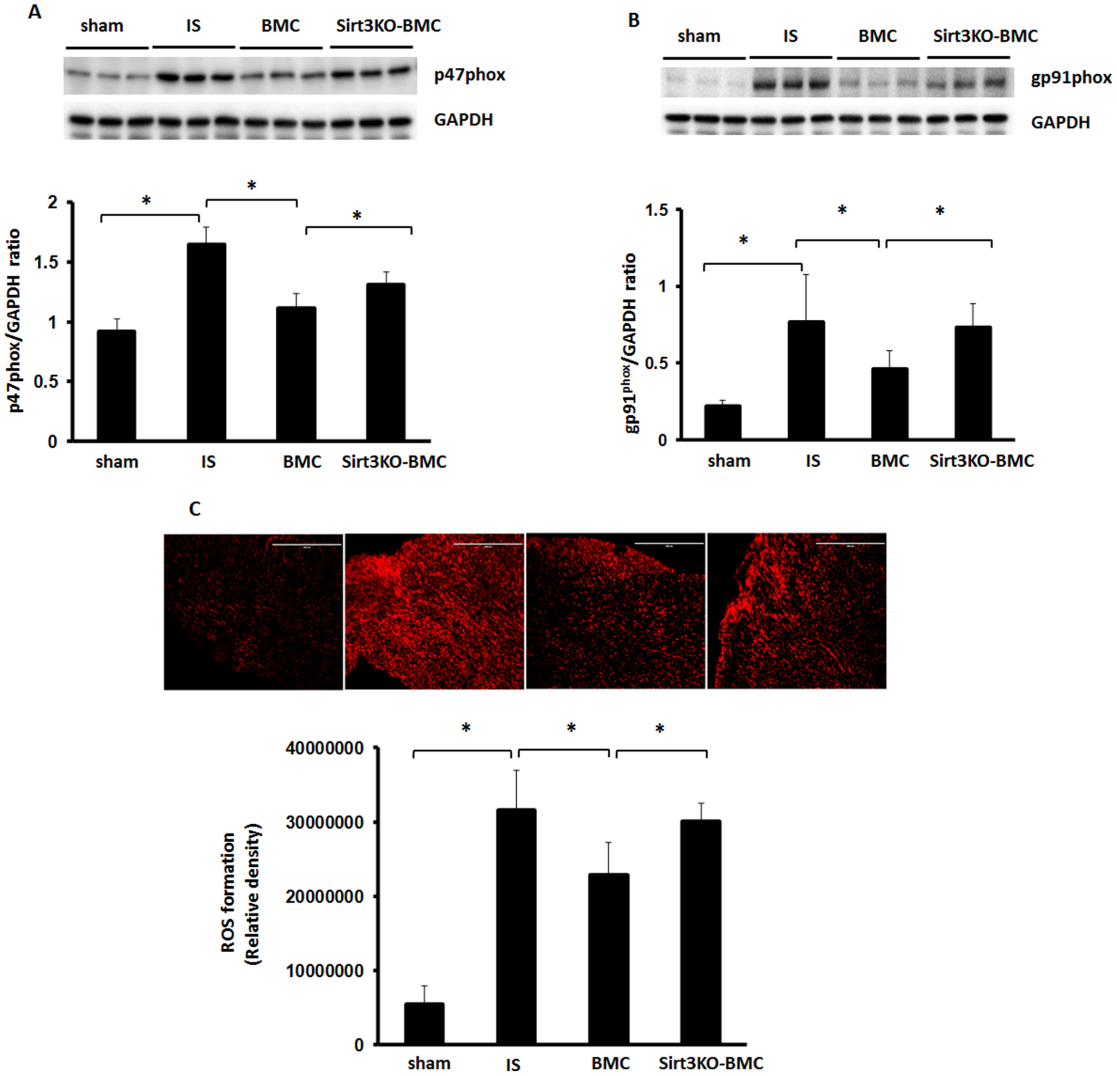
Loss of Sirt3 in BMCs increases ROS formation in post-MI mice. **A and B**. Western blot analysis revealing that BMC treatment resulted in a significant downregulation of p47^phox^ (A) and gp91^phox^ (B) expression compared to post-MI mice. Sirt3KO-BMC treatment did not inhibit expression of p47^phox^ and gp91^phox^ compared to BMC treatment. n = 6 mice, *p<0.05. **C**. Quantitative analysis of ROS formation by DHE staining revealing that BMC treatment resulted in a significant suppression of ROS formation compared to post-MI mice. Myocardial ROS formation was significantly higher in Sirt3KO-BMC treated mice than BMC treated mice. n = 5 mice; *p<0.05.

To determine whether Sirt3 is involved in BMC-mediated suppression of ROS formation, NADPH oxidase subunits p47^phox^ and gp91^phox^ expression was examined in the hearts of post-MI mice. BMC treatment led to a significant reduction of NADPH oxidase subunits p47^phox^ and gp91^phox^ expression in ischemic hearts (**Fig 4 A and B**). This was accompanied by a significant reduction of ROS formation in post-MI mice (**Fig 4 C**). In contrast, treatment with Sirt3KO-BMCs did not inhibit p47^phox^ and gp91^phox^ expression in post-MI mice (**Fig 4 A and B**). ROS formation was significantly elevated in Sirt3KO-BMCs + MI mice when compared to WT-BMCs + MI mice (**Fig 4C**).

### Loss of Sirt3 in BMCs impairs angiogenesis in post-MI

**Figure 5:**
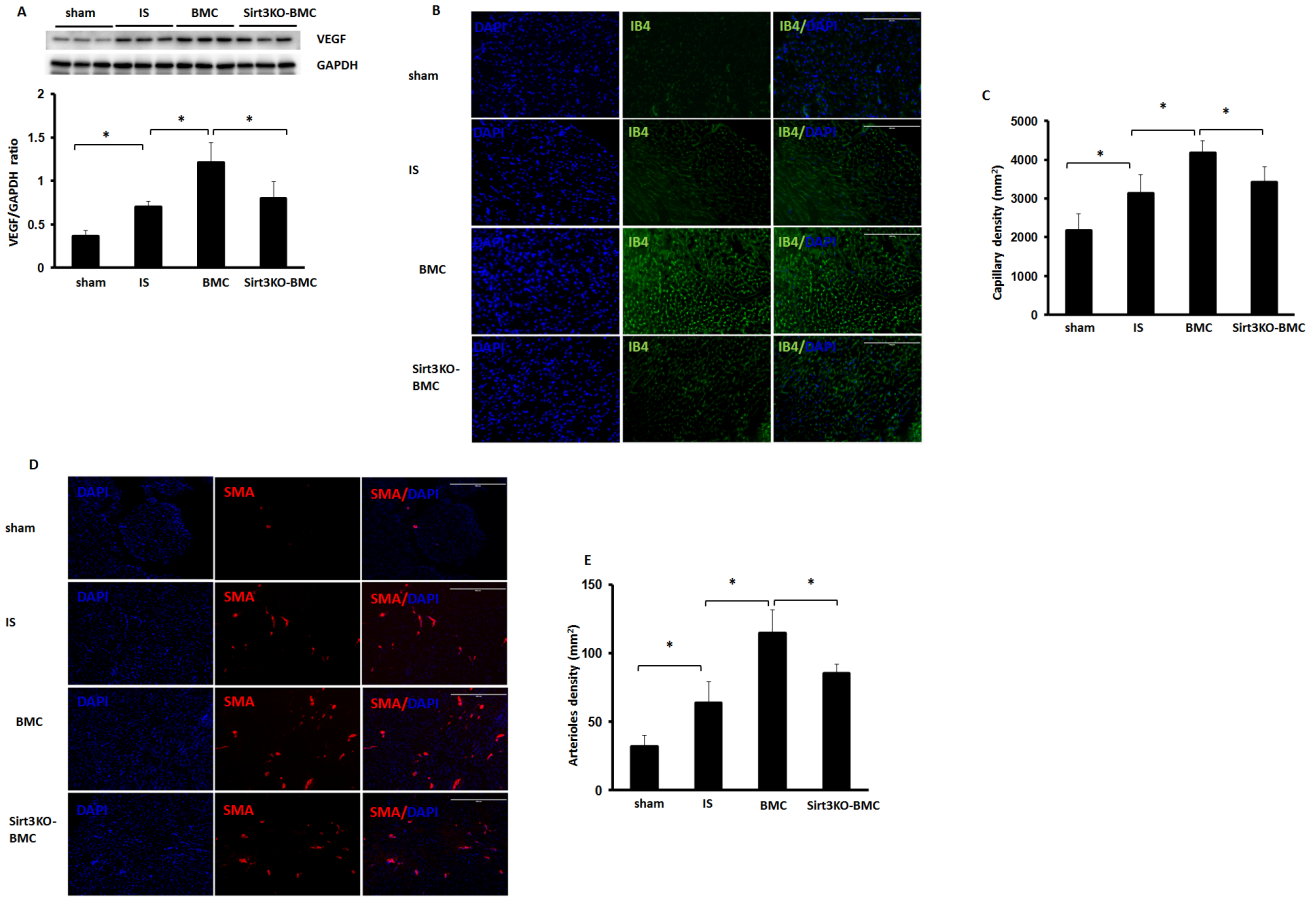
Loss of Sirt3 blunts BMC-induced VEGF expression and angiogenesis. **A**. Western blot analysis showing that VEGF expression was significantly reduced in Sirt3KO-BMC treated mice compared to BMC treated mice. n = 6 mice; *p<0.05. **B and C**. Myocardial ischemia significantly increased myocardial capillary density by IB4 staining (green, 10x). BMC treatment significantly increased capillary formation compared to post-MI mice. Myocardial capillary density was significant reduced in Sirt3KO-BMC treated mice compared to BMC treated mice. n = 5 mice; *p<0.05. **D and E**. BMC treatment significantly increased myocardial arteriole density by SMA staining (Red, 10x) in post-MI mice. Myocardial arteriole density was significantly decreased in post-MI mice treatment with Sirt3KO-BMCs compared to mice treated with BMCs. n = 5 mice; *p<0.05.

To elucidate whether Sirt3 in the BMCs is necessary for the myocardial angiogenesis, VEGF, capillary and arteriole densities were examined in post-MI mice. BMC treatment significantly increased VEGF expression in the hearts of post-MI mice. VEGF expression was significantly decreased in Sirt3KO-BMCs + MI mice compared with WT-BMCs + MI mice (**Fig 5A**). BMC treatment further significantly increased capillary and arteriole densities in the border zone of ischemic hearts. However, myocardial capillary and arteriole densities were not significantly increased in Sirt3KO-BMCs + MI mice when compared with post-MI mice (**Fig 5 B-E**).

### Sirt3 is necessary for BMC-mediated anti-apoptosis in ischemic hearts

**Figure 6:**
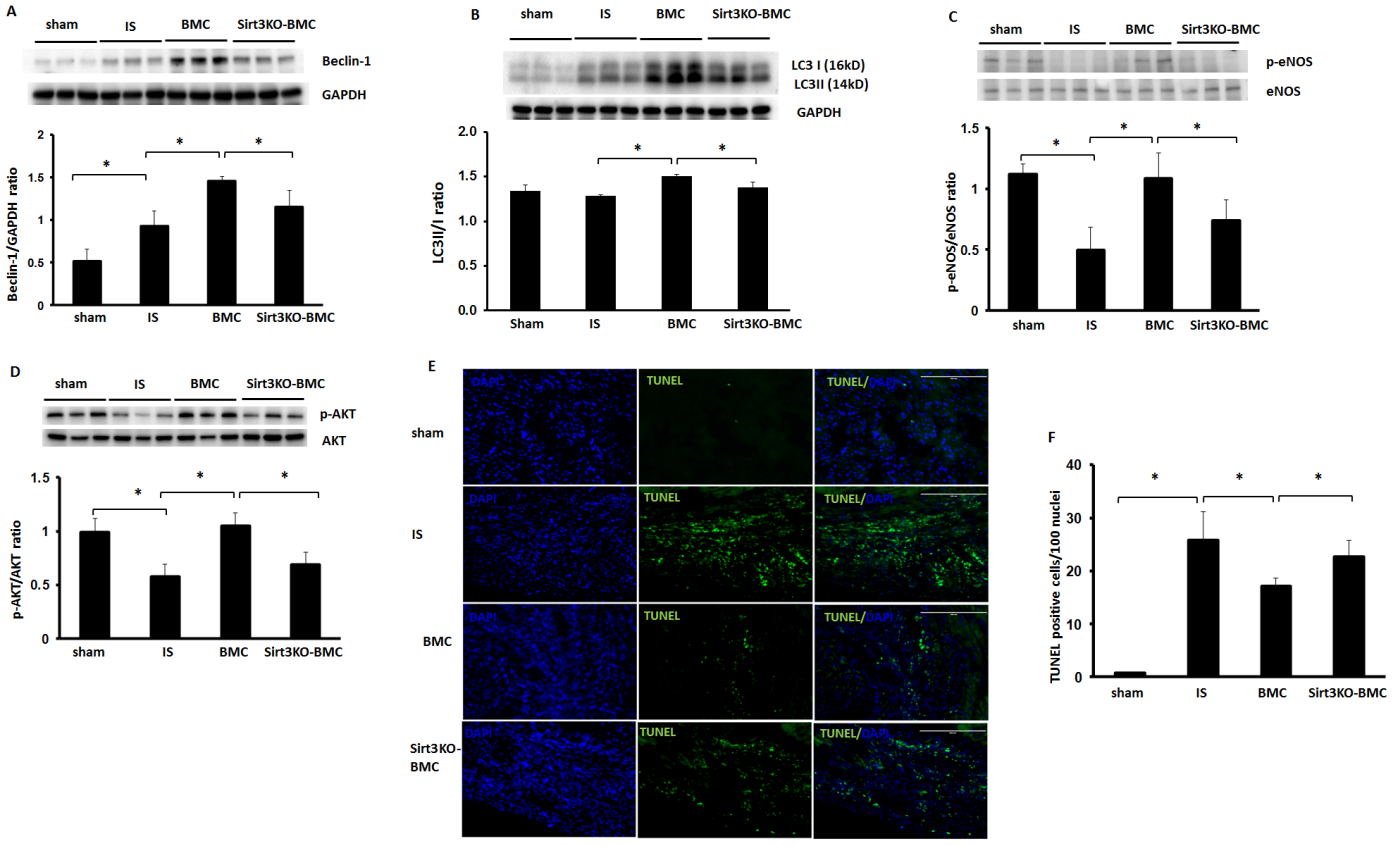
Loss of Sirt3 in BMCs increases apoptosis in post-MI mice. **A and B**. Western blot analysis demonstrating that treatment of post-MI mice with BMCs resulted in a significant increase in autophagy gene beclin-1 expression (A) and LC3-II/I ratio (B). Beclin-1 expression and LC3-II/I ratio were significantly decreased in Sirt3KO-BMC treated mice compared to BMC treated post-MI mice. n = 6 mice; *p<0.05. **C and D**. Western blot analysis demonstrating that BMC treatment significantly increased phosphorylation levels of eNOS (C) and Akt (D) in post-MI mice. The phosphorylation levels of Akt and eNOS were significantly decreased in Sirt3KO-BMC treated mice compared to BMC treated mice. n = 6 mice; *p<0.05. **E and F**. Apoptotic cells in the infarcted area of the left ventricle were identified by TUNEL staining (green, 10x). Treatment of post-MI mice with BMCs significantly decreased TUNEL^+^ cells in ischemic area. TUNEL^+^ cells were significantly increased in Sirt3KO-BMC treated mice when compared with BMC treated mice. n = 5 mice; *p<0.05.

To further elucidate whether Sirt3 in the BMCs is also necessary for the cardiac repair of BMC therapy, myocardial autophagy gene expression, apoptosis, fibrosis and hypertrophy were examined in post-MI mice. Treatment with BMCs led to a significant increase in autophagy gene beclin-1 expression and elevation of LC3-II/I ratio in post-MI mice (**Fig 6A and B**). Beclin-1 expression and LC3-II/I ratio was significantly decreased in Sirt3KO-BMCs+MI mice when compared with WT-BMCs + MI mice. BMC treatment significantly elevated phosphorylation levels of Akt and eNOS in post-MI mice (**Fig 6C and D**). The phosphorylation levels of Akt and eNOS were significantly reduced in Sirt3KO-BMCs + MI mice compared to BMCs+ MI mice. BMC treatment furthermore significantly reduced cardiac apoptosis, but Sirt3KO-BMC treatment did not affect cell apoptosis in post-MI mice (**Fig 6 E and F**).

### Loss of Sirt3 in BMCs limits BMC-mediated cardiac repair and functional recovery

**Figure 7:**
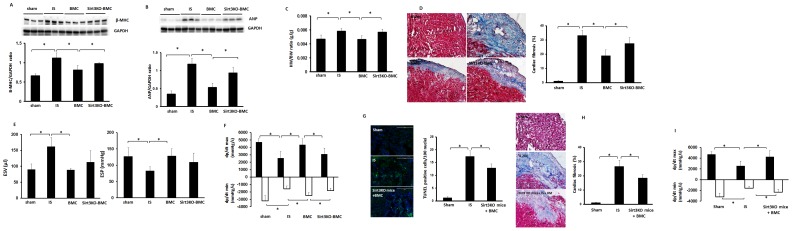
Loss of Sirt3 abolished BMC-mediated cardiac repair in post-MI mice. **A and B**. Western blot analysis showing that treatment of post-MI mice with BMCs significantly reduced hypertrophic marker β-MHC (A) and ANP (B) expression. Treatment of post-MI mice with Sirt3KO-BMC failed to suppression of β-MHC and ANP expression compared to BMC treated mice. n = 6 mice, *p<0.05. **C**. BMC treatment significantly reduced HW/BW ratio in post-MI mice. Treatment with Sirt3KO-BMCs failed to significant reduction of HW/BW ratio compared to BMC treated mice. n = 6 mice, *p<0.05. **D**. Representative images of cardiac fibrosis in the infarction zone and quantitative analysis of fibrotic area in mice (Masson's trichrome). BMC treatment significantly reduced the area of cardiac fibrosis. Sirt3KO-BMC treatment significantly increased cardiac fibrosis area compared to BMC treated mice. n = 5 mice; *p<0.05. **E**. The end-systolic volume (ESV) was significantly increased in post-MI mice. BMC treatment significantly decreased ESV. The end-systolic pressure (ESP) was decreased in post-MI mice. Treatment with BMCs significantly increased ESP whereas treatment of post-MI mice with Sirt3KO-BMC had little effects on ESV and ESP. n = 5–7 mice, *p<0.05. **F**. BMC therapy led to a significant improvement of maximum +dP/dt and minimum -dP/dt pressures compared to control post-MI mice. Sirt3KO-BMC treatment failed to improve maximum +dP/dt and minimum -dP/dt pressures in post-MI mice. n = 5–7 mice,*p<0.05. **G**. Treatment of Sirt3KO post-MI mice with WT-BMCs significantly reduced TUNEL^+^ cells in ischemic area. Apoptotic cells in the infarcted area of the left ventricle were identified by TUNEL staining (green, 10x). n = 6 mice; *p<0.05. **H**. Treatment of Sirt3KO post-MI mice with WT-BMCs significantly reduced the area of cardiac fibrosis (Masson's trichrome). n = 5 mice; *p<0.05. **I**. Treatment of Sirt3KO post-MI mice with WT-BMCs significantly improved maximum +dP/dt and minimum -dP/dt pressures in post-MI mice. n = 5–6 mice,*p<0.05.

In comparison with control post-MI mice, the HW/BW ratio and expression of hypertrophic gene β-MHC and ANP were significantly reduced in the BMCs + MI mice (**Fig 7 A, B, C**). Sirt3KO-BMC treatment did not suppress cardiac hypertrophy compared with BMC treatment (**Fig 7 A, B, C**). BMC treatment also significantly suppressed cardiac fibrosis formation whereas Sirt3KO-BMC treatment had little effects (**Fig 7D**).

After 28 days of myocardial ischemia, post-MI mice exhibited a significant elevation of end-systolic volume (ESV) and reduction of end-systolic pressure (ESP) (**Fig 7E**). The post-MI mice also showed a significant decline in +dp/dtmax and -dp/dtmin pressures compared to non-ischemic sham controls (**Fig 7F**). BMC treatment resulted in a significant decrease in ESV and a dramatic improvement of ESP, +dp/dtmax and -dp/dtmin pressures in post-MI mice. Sirt3KO-BMC treatment had little effects on the improvement of these parameters when compared with WT-BMC treatment (**Fig 7E and F**). In contrast, treatment of Sirt3KO post-MI mice with WT-BMCs resulted in a significant reduction of cardiac apoptosis and cardiac fibrosis formation (**Fig 7 G and H**). This was accompanied by a significant improvement of cardiac function in Sirt3KO post-MI mice (**Fig 7I**).

## Discussion

Our present study demonstrates that loss of Sirt3 in EPCs reduced angiogenic growth factor expression and angiogenic capacity. Loss of Sirt3 in EPCs increased ROS formation and promoted cell apoptosis *in vitro*. Furthermore, loss of Sirt3 in BMCs abolished BMC therapy mediated protective effects and limited cardiac repair in post-MI mice. Our study suggests that Sirt3 in BMCs is necessary for the protective effects of stem cell therapy in post-MI.

Sirt3 has been reported to be a major mitochondrial deacetylase in human [2], [32], [33]. Previous studies show that Sirt3 exists in the mitochondria of the heart [34], [35]. Our recent study indicates a critical role of Sirt3 in apelin-overexpressing BMC-mediated improvement of angiogenesis and cardiac function in post-MI mice [20]. In present study, we show that treatment with BMCs resulted in a significant increase in Sirt3 expression in post-MI mice. We then further investigated if BMC treatment improved Sca1^+^/c-kit^+^ progenitor cells in ischemic hearts. Our data demonstrated that the number of Sca1^+^/c-kit^+^ progenitor cells in ischemic hearts was increased at 14 days of post-MI. Injection of BMCs significantly increased the number of Sca1^+^/c-kit^+^ cells and promoted cardiac repair at ischemic area in post-MI mice. Intriguingly, the number of Sca1^+^/c-kit^+^ cells was significantly reduced in Sirt3KO-BMC treatment. This was accompanied by a significant decline of cardiac function in post-MI mice. A recent study demonstrated that there was no difference in the number of BM derived HSCs (Lin^−^, c-kit^+^, sca1^+^) between WT and Sirt3 KO mice [21]. Consistent with this study, we did not found any GFP^+^-BMCs or GFP^+^-Sirt3KO-BMCs in hearts of post-MI mice after 14 and 28 days of BMC treatment, suggesting that injected BMCs had not differentiated into Sca1^+^/c-kit^+^ cells in ischemic hearts and Sca1^+^/c-kit^+^ cells was not coming from injected BMCs. CXCR-4 has been identified as a key mediator that regulates vascular progenitor cell homing into the ischemic area and contributes to improvement of cardiac function after MI [36]. CXCR-4 has been shown to protect the heart after myocardial infarction via promoting stem cell recruitment [36]–[38]. We therefore speculated that these increased Sca1^+^/c-kit^+^ cells may be recruited to ischemic area due to releasing CXCR-4 after BMC treatment. This notion was confirmed by our data that the basic levels of CXCR-4 were significantly reduced in Sirt3KO-EPCs. Our data suggest that impairment of CXCR-4 expression in EPCs and reduction of number of Sca1^+^/c-kit^+^ stem cell in infarcted area may be responsible for impairment of cardiac repair in Sirt3KO-BMC treatment in post-MI mice.

The present study provides evidence that basal Sirt3 activity in EPC is required for the protective effects afforded by BMC therapy in post-MI. Myocardial ischemia has been shown to induce rapid mobilization of bone marrow derived vascular progenitor cells from the bone marrow niches [39], [40]. BMCs are recruited into the sites of infarcted area and promote cardiac repair in the infarcted hearts [16], [17]. BMCs have been shown to promote cardiac repair and improve functional recovery of post-MI; however, the molecular mechanisms by which BMCs promotes cardiac repair are incompletely understood. Our data, for the first time, showed that Sirt3 levels were reduced in ischemic hearts. Recently studies have directly linked loss of Sirt3 contributing to the ROS formation. Sirt3-deficient cells subjected to metabolic stress lead to a significant increase in ROS formation [41]. Furthermore, cardiomyocytes cultured from Sirt3KO mice show increased ROS production. Sirt3 protects cardiomyocytes from oxidative stress-mediated cell death [15]. Therefore, reduction of Sirt3 levels in the ischemic hearts may contribute to increased ROS formation and apoptosis. In line with these studies, our present data also showed that loss of Sirt3 in EPCs enhanced ROS formation and increased stress-induced cell apoptosis. Furthermore, treatment with NADPH oxidase inhibitor reduced Sirt3KO-EPC apoptosis *in vitro*. Overexpression of Sirt3 protected EPCs against stress-induced cell apoptosis. Our data further showed that intramyocardial delivery of BMCs in infarcted area led to a significant increase in Sirt3 expression. Moreover, treatment with BMCs resulted in a significant reduction of NADPH oxidase p47^phox^ and gp91^phox^ expression and ROS formation. In contrast, loss of Sirt3 in BMCs significantly blunted BMC therapy mediated upregulation of Sirt3 and suppression of ROS formation. This was accompanied by a significant increase in myocardial apoptosis in post-MI mice. Taken together, our data suggest that a critical role of Sirt3 in the regulation of ROS formation and apoptosis in the ischemic heart and that increased ROS formation and apoptosis in Sirt3KO-EPCs maybe also contribute, at least in part, to the failure of Sirt3KO-BMC treatment in post-MI.

Aging bone marrow cells have been shown to fail to promote cardiac angiogenesis and improve cardiac function [42]. Both experimental and clinical studies demonstrated that aging interferes with bone marrow derived progenitor cell functions [43]–[45]. ROS formation is increased in HSCs with age. This is accompanied by an increased HSCs apoptosis and impairment of self-renewal capacity. Sirt3 expression is significantly reduced with the aging in skeletal muscle [9]. A recent study also shows a significant reduction of Sirt3 levels with age or stress in HSCs. Overexpression of Sirt3 in HSCs from old mice improves regenerative capacity of aged HSCs [21]. In this study, we hypothesized that loss of Sirt3 in BM stem cells; similar as aged BM derived HSCs, fails to improve angiogenesis and cardiac repair in post-MI. To substantiate this notion, we first compared the expression of angiogenic growth factor and angiogenesis between WT-EPCs and Sirt3KO-EPCs *in vitro*. Our data showed that loss of Sirt3 in EPCs reduced VEGF and VEGFR2 expression. Moreover, treatment with NADPH oxidase inhibitor or overexpression of Sirt3 rescued impaired VEGF and VEGFR2 expression. In addition, the basal proliferation and angiogenic capacities were significantly reduced in Sirt3KO-EPCs. Our study *in vivo* further confirmed that BMC treatment increased VEGF expression and elevated phosphorylation levels of eNOS and Akt. This was accompanied by increased myocardial vascular densities and improved cardiac function in post-MI mice. In contrast, knockout of Sirt3 in BMCs reduced BMC-mediated VEGF expression and neovascularization. Furthermore, loss of Sirt3 in BMCs abolished BMC-mediated cardiac repair and improvement of cardiac function in post-MI mice. These findings indicate that lack of Sirt3 and increased ROS formation in aged EPCs maybe contribute to the failure of aged BMC treatment in post-MI.

Autophagy is a dynamic process of intracellular bulk degradation in which cytosolic proteins and organelles are fused with lysosomes for degradation. Under stressed conditions, autophagy selectively removes damaged mitochondria which prevent activation of apoptotic machinery [46], [47]. Overexpression of autophagy gene beclin-1 has been shown to protect cardiac myocyte against ischemia/reperfusion injury [48]. Autophagy has been shown to have a protective role in the heart following myocardial ischemia/reperfusion *in vivo* [49]–[52]. Our previous study indicates that Sirt3 is necessary for apelin-BMC therapy-mediated upregulation of autophagy. Loss of Sirt3 attenuates apelin-induced autophagy gene marker beclin-1 and LC3-I/II expression [20]. In the present study, we demonstrate that treatment with BMCs led to a significant increase in autophagy gene beclin-1 expression and elevation of LC3-II/I ratio. These are accompanied by a dramatic reduction of cardiac apoptosis. However, treatment with Sirt3KO-BMC had no effect on LC3-II/I ratio and Beclin-1 expression in post-MI mice. This was associated with a significant higher number of apoptotic cells in ischemic hearts. Our studies revealed a novel molecular mechanism of BMC stem cell therapy which stem cells may attenuate apoptosis via regulation of autophagy in post-MI. Autophagy also has been shown to promote stem cell generation and differentiation. Inhibition of autophagy reduces stem cell self-renewal and differentiation [53]–[57]. A recent study further underscores the important role of autophagy in EPC survival, proliferation and differentiation. Inhibition of autophagy reduces proliferation and differentiation of EPCs. In contrast, increasing autophagy enhances EPC survival under hypoxic conditions [53]. Our data showed that loss of Sirt3 reduced autophagic gene LC3-II levels whereas overexpression of Sirt3 or treated with NADPH oxidase inhibitor increased LC3-II levels in EPCs. Overexpression of Sirt3 further attenuated EPCs apoptosis. These data suggest that reduction of autophagy may be contributed to the higher apoptosis of Sirt3KO-EPCs. Although Sirt3 has been shown to rejuvenate HSCs [21], so far, it remains unanswered what is necessary for Sirt3 to complete its rejuvenation. In addition, if Sirt3-induced HSCs rejuvenation requires to removing additional damaged organelles such as mitochondria via regulation of autophagy remains unknown. Further studies are warranted to elucidate the molecular mechanisms by which Sirt3 regulates autophagy in stem cell rejuvenation.

In summary, the current study provides evidence that basal levels of Sirt3 in stem cells contribute the therapeutic effects of BMCs in post-MI mice. Since the levels of Sirt3 were reduced in aging and aged stem cells, our findings implicate that reduced levels of Sirt3 may contribute to the failure of BMC therapy in aging patients. Our findings further suggest that augmentation of Sirt3 activity in stem cells may represent a novel therapeutic approach for the improvement of stem cell therapy for the ischemic heart diseases.
